# A novel murine model for sporadic, malignant peripheral nerve sheath tumors, driven by *Braf^V600E^* and *Pten* loss

**DOI:** 10.1242/dmm.052471

**Published:** 2025-11-28

**Authors:** Julien Debbache, Myriam Gwerder, Elisabeth Rushing, Lukas Sommer

**Affiliations:** ^1^ University of Zurich, Institute of Anatomy, Winterthurerstrasse 190, 8057 Zürich, Switzerland; ^2^ University Hospital of Zurich, Institute of Neuropathology, Schmelzbergstrasse 12, 8091 Zürich, Switzerland

**Keywords:** MPNST, Cancer, Mouse model, *Braf*, *Pten*

## Abstract

Malignant peripheral nerve sheath tumors (MPNSTs) are aggressive sarcomas with limited therapeutic options. Here, we present a novel sporadic murine model of *Nf1* wild-type MPNST, driven by conditional expression of oncogenic *Braf^V600E^* and loss of *Pten* in the glial lineage using the *Plp1::CreER^T2^* driver. This model allows for highly penetrant and rapid tumor induction through spontaneous formation, localized initiation, or cell transplantation. Comparative analysis with *Tyr::CreER^T2^*-driven melanoma revealed striking phenotypic divergence despite shared genetic alterations, underscoring the importance of the cell of origin in shaping tumor identity. In this system, MPNST cells show refractory capacities to induce melanocytic trans-differentiation upon melanoma-promoting signaling cues, such as canonical Wnt signaling gain of function or increased of levels of the epigenetic mark H3K27Me3 upon Ezh2 gain of function. Our findings emphasize the significance of lineage context in tumor initiation and provide a foundation for future mechanistic and therapeutic studies.

## Teaser

**Summary:** This study presents a versatile murine model of sporadic MPNST, highlighting how the cell of origin shapes tumor phenotype under shared genetic alterations and offering a foundation for lineage-informed cancer research.

## INTRODUCTION

Both Schwann cells and melanocytes derive from a common developmental structure, the neural crest (NC). Multiple lines of evidence suggest that, in some cases, these two lineages share the same common progenitor until very late in their respective lineage specification (Colombo et al., 2022; Kaucka et al., 2021). Even in the adult organism, Schwann cells and melanocytes remain in close proximity in certain tissues, such as the bulge region of the hair follicle (HF), where Sox10^+^ Ngfr^+^ stem cell-like populations have been characterized (Wong et al., 2006). This developmental and spatial relationship could have significant implications for tumorigenesis, as both cell types can give rise to aggressive malignancies – melanoma and malignant peripheral nerve sheath tumors (MPNSTs), respectively.

Melanoma, a rare but highly lethal form of skin cancer, is one of the best-characterized cancer types. Multiple model organisms, induction methods, and genetic modifications have provided valuable insights into the mechanisms of disease initiation and progression (Rebecca et al., 2020). A key molecular feature of melanoma is the frequent occurrence of activating mutations in the MAPK pathway, particularly in *BRAF* (most commonly the oncogenic *BRAF^V600E^* mutation), which has been successfully targeted with inhibitors such as vemurafenib (Kim and Cohen, 2016; Wellbrock and Hurlstone, 2010). These models have played a crucial role in the development of targeted therapies, significantly improving patient outcomes.

In contrast, MPNSTs are aggressive, treatment-refractory sarcomas, most of which arise from the Schwann cell lineage (Cortes-Ciriano et al., 2023; Gupta et al., 2008). The vast majority of MPNSTs develop in individuals with germline mutations in the neurofibromatosis type 1 (*NF1*) gene, a MAPK suppressor, accounting for 50-60% of cases. In addition, ∼20% of all individuals with MPNST harbor sporadic *NF1* mutations (Kaplan et al., 2018; Mohamad et al., 2021). Owing to the strong association with *NF1* loss, most preclinical MPNST models have been developed in NF1-deficient systems, either alone or combined with conditional deletion of additional tumor suppressors such as *Tp53* (also known as *Trp53*) or *Cdkn2a* (Mohamad et al., 2021). However, these *in vivo* models often suffer from low penetrance and relatively long tumor latency, with median survival ranging from 4 to 6 months, which limits their utility for studying early tumorigenic events and hampers efficient preclinical testing. Further, a subset of individuals with *NF1* wild-type MPNST (3-12% depending on the study) have been reported to harbor *BRAF* mutations, among which *BRAF^V600E^* is the most frequent, similar to observations in melanoma and additional epithelial cancers (Hirbe et al., 2014; Kao et al., 2022; Kaplan et al., 2018). Despite its known oncogenic potential, the role of BRAF in MPNST pathogenesis remains largely unexplored, and there are currently no preclinical models specifically designed to study BRAF-driven MPNSTs.

Given the success of BRAF inhibitors in melanoma and other malignancies, establishing a model for BRAF-driven MPNST is crucial for evaluating targeted treatment strategies. Additionally, gain-of-function alterations in the PTEN-PI3K-AKT signaling pathway, observed in up to 46% of MPNSTs may further cooperate with BRAF to drive malignancy, necessitating dedicated models that recapitulate these molecular interactions (Gregorian et al., 2009; Kaplan et al., 2018; Keng et al., 2012).

Developing a preclinical BRAF-driven MPNST model would provide a valuable tool for dissecting the molecular underpinnings of this tumor subset and for testing proven, targeted therapies. Such a model could help determine whether MPNST patients harboring *BRAF* mutations might benefit from MAPK-targeted therapies, either alone or in combination with PI3K-AKT inhibitors. Addressing this gap in available models is imperative for expanding treatment options for MPNST patients and improving clinical outcomes in this highly aggressive cancer type.

## RESULTS

### Dual MAPK PI3K activation in glial cells confer tumorigenesis reminiscent of MPNSTs

**Fig. 1.**
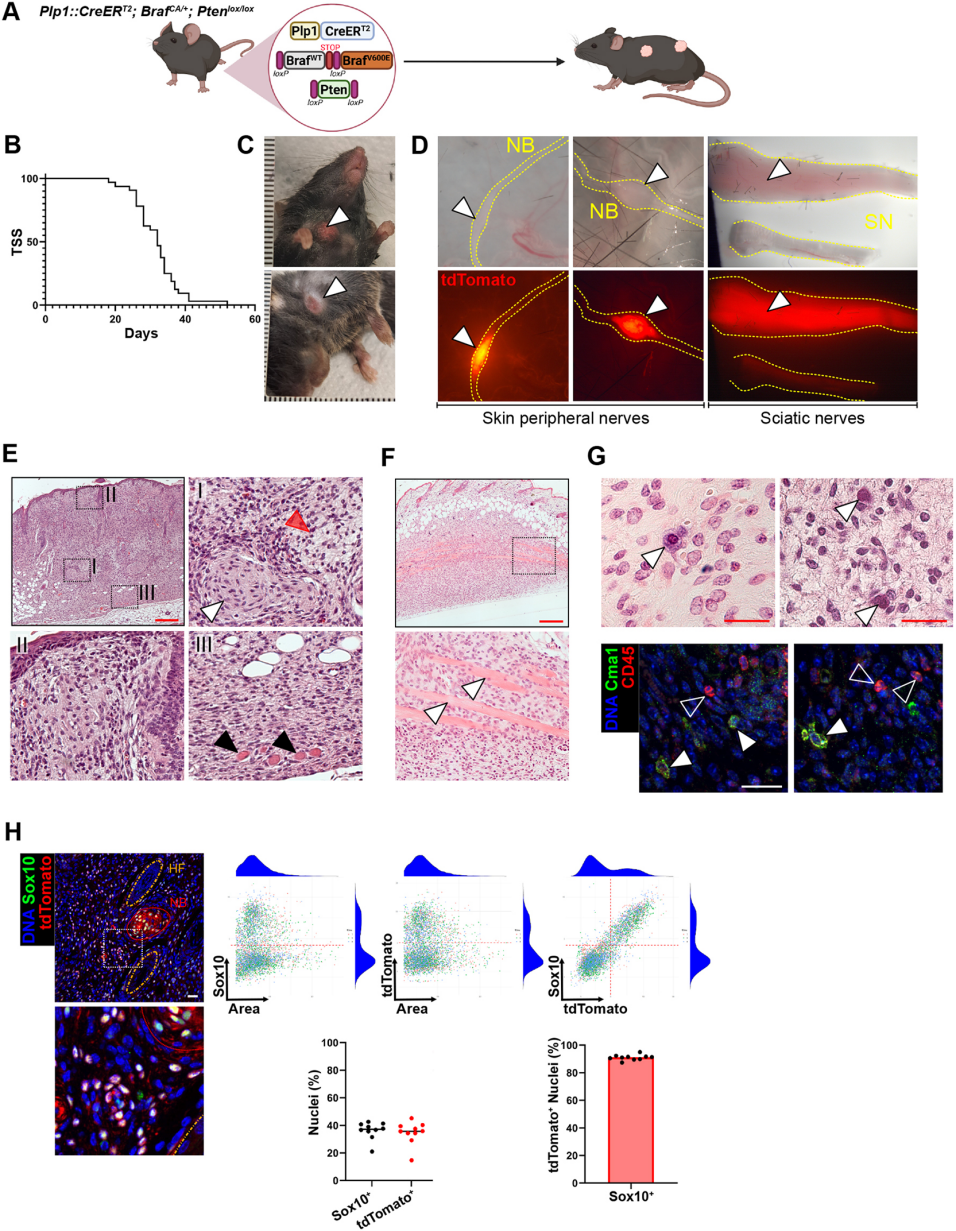
***Plp1*-driven oncogenic BRAF^V600E^ expression and concomitant *Pten* cKO gives rise to MPNST-like tumors in mice.** (A) Allelic composition of the genetic model used to drive Cre-mediated MAPK and PI3K activation in peripheral glia. Despite its supposed TM-dependent induction, untreated mice develop tumors over time. (B) Kaplan–Meier curve representing the tumor-specific survival (TSS) of *Plp1::CreER^T2^*; *Braf^CA/+^*; *Pten^lox/lox^* animals without TM treatment (median survival=32 days; *n*=32 mice). (C) Representative amelanotic skin tumors (arrowheads) appearing spontaneously in the compound transgenic animals. (D) Macroscopic images of tumors associated with peripheral nerves in the skin and with sciatic nerves. *R26^LSL-tdTomato^* (*tdTomato^lox^*)-bearing mice show fluorescence signal in recombined tumor tissue. Dotted lines outline subdermal nerve bundles (NB) or dissected sciatic nerves (SN). Arrowheads indicate focal expansion of tdTomato+ cells. (E) Hematoxylin-Eosin staining of spontaneous intradermal tumors. Inset I: White arrowhead indicates imbedded hypertrophic nerves, red arrowhead loose extracellular matrix. Inset II: Subepidermal region with immune infiltrates. Inset III: Subdermal region. Black arrowheads indicate tumor cell-infiltrated panniculus carnosus muscle bundles. (F) Hematoxylin-Eosin staining of spontaneous intradermal tumors. White arrowheads indicate panniculus carnosus muscle bundles. Bottom image is a high-magnification view of the boxed area above. (G) Hematoxylin-Eosin staining and corresponding immunofluorescence (IF) staining for mast cell chymase (Cma1) and CD45. White arrowheads indicate dispersed Cma1^+^/CD45^+^ mast cells, unfilled arrowheads Cma1^−^/CD45^+^ cells. (H) Representative IF images showing Sox10^+^/tdTomato^+^ tumor cells alongside the unlabeled stromal compartment and distributions of segmented nuclei based on object size (area) and single-channel normalized fluorescence intensity and corresponding proportions of Sox10^+^ (normalized intensity >2.5) and tdTomato^+^ (normalized intensity >3). Hair follicles (HFs) are outlined with orange dashed lines, and nerve bundles (NB) with red dotted lines. *n*=10. Scale bars: 250 µm (E,F), 25 µm (G,H).

Unlike *Nf1* depletion, embryonic expression of Braf^V600E^ in the NC is lethal (Dhomen et al., 2010); therefore, we implemented a temporally controllable expression system, as described by Dankort et al. for the generation of a genetic melanoma model with a concomitant MAPK PI3K activation. In their model, melanocyte-specific expression of Braf^V600E^ (*Braf^CA^* allele) and depletion of *Pten* (*Pten^lox^* allele) are achieved using the *Tyr::CreER^T2^* transgene, a tamoxifen (TM)-inducible Cre recombinase (Dankort et al., 2009). To achieve efficient glial recombination in peripheral nerves, we opted for the *Plp1::CreER^T2^* driver (Fig. 1A) (Leone et al., 2003), previously described to promote MPNST in *Nf1* cKO models (Brosseau et al., 2018; Le et al., 2011).

In the absence of TM induction, however, *Plp1::CreER^T2^; Braf^CA/+^; Pten^lox^^/lox^* mice developed amelanotic skin tumors with an extremely short latency, likely due to partial leakage of *CreER^T2^* recombinase expression before induction (Fig. 1B,C). In mice carrying in addition the *R26-tdTomato^LSL^* reporter allele (*tdtomato^lox^*), fluorescently traced focal enlargements of peripheral nerves were observed in the skin (Fig. 1D). Additionally, upon euthanasia internal tumor lesions associated with nerves were identified. A subset of animals (3/32) exhibited neuromuscular impairments that rapidly progressed to complete limb paralysis. Affected limbs exhibited marked enlargement and widespread tdTomato expression along sections of their sciatic nerve, in contrast to the contralateral, unaffected side (Fig. 1D).

The tumors exhibited a heterogeneous architecture, composed of discohesive oval to spindle-shaped cells within a variably dense extracellular matrix, interspersed with collagen deposits and traversed by hypertrophic nerves. Tumors extended through the full thickness of the skin, from the epidermis to the subcutis, and appeared to arise from or infiltrate innervated structures, including HFs and the subcutaneous muscle layer (panniculus carnosus) (Fig. 1E,F). Additionally, mast cells with abundant cytoplasmic granules, positive for leukocyte common antigen (CD45; Ptprc) and mast cell chymase (Cma1) were scattered throughout the tumors, a characteristic feature of MPNSTs (Fig. 1G). Unlike typical solid tumors, immunofluorescence staining for the NC marker Sox10 and the genetic tracer tdTomato showed that most cells were non-recombined stromal cells, while the tdTomato^+^ tumor cell fraction, just under 40% of all cells, consistently expressed Sox10 (>90%) (Fig. 1H).

**Fig. 2.**
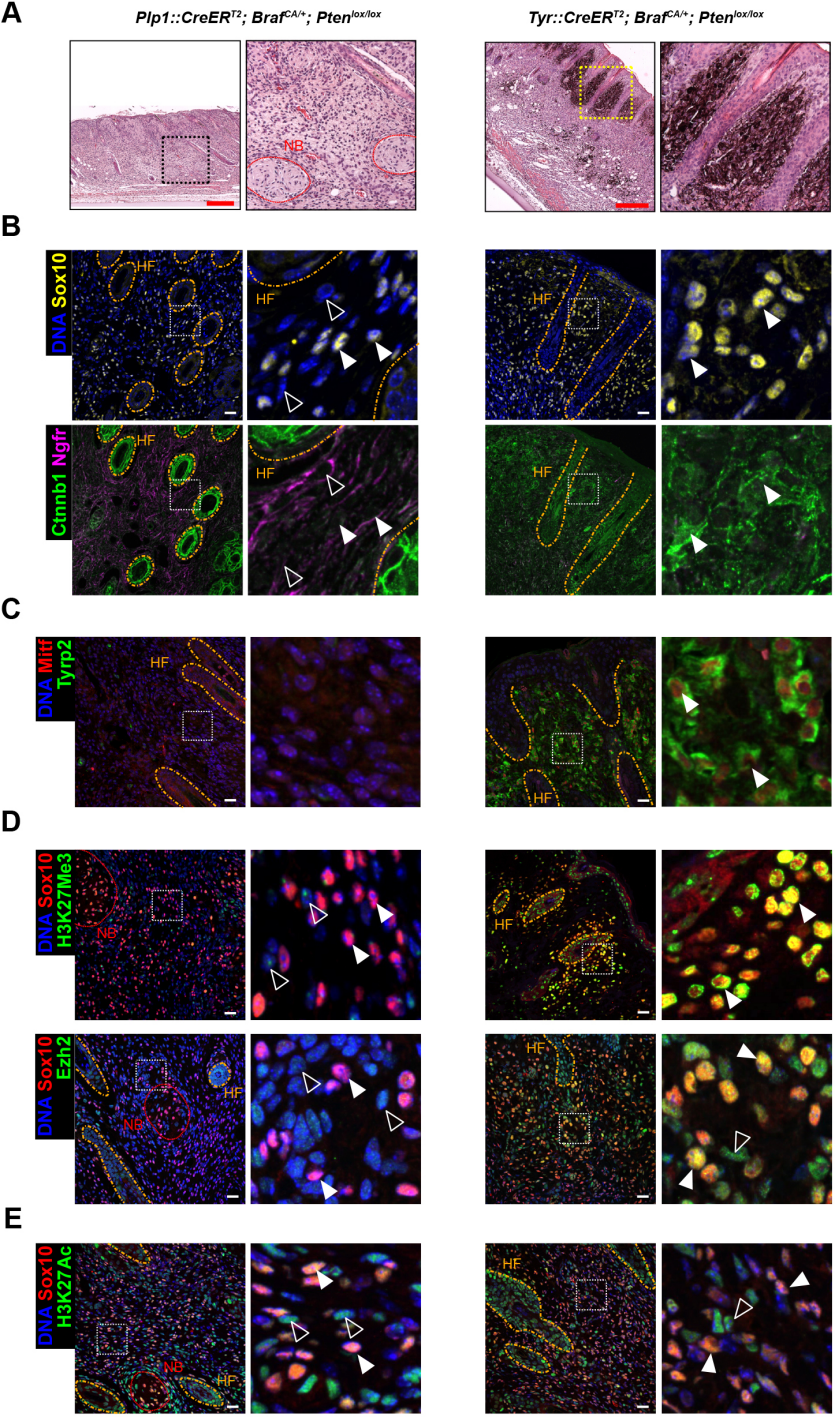
***Plp1*-driven MPNSTs diverge from *Tyr*-driven melanomas.** (A-E) Comparative histology of spontaneous *Plp1::CreER^T2^*; *Braf^CA/+^*; *Pten^lox/lox^* and 4-OHT-induced *Tyr::CreER^T2^*; *Braf^CA/+^*; *Pten^lox/lox^* skin tumors with overview and high-magnification views of the boxed areas. (A) Hematoxylin-Eosin staining of a section of *Plp1*-driven MPNST-like tumor and corresponding *Tyr*-driven melanoma. (B-E) IF staining for Sox10, Ctnnb1 and Ngfr (B), the melanocytic markers Mitf and Tyrp2 (C; arrowheads indicate Mitf^+^/Tyrp2^+^ cells), and Sox10 in combination with the PRC2 activity marker H3K27Me3 and the PRC2 component Ezh2 (D), or with the histone activation mark H3K27Ac (E). Nuclei are counterstained with Hoechst 33342. White arrowheads indicate tumor cells; unfilled arrowheads, stromal cells. Orange dashed lines label HFs, red dotted lines label nerve bundles (NBs). Images are representative of *n*>10 (A−D), *n*=5 (E) samples. Scale bars: 25 µm.

In contrast to melanomas derived from *Tyr::CreER^T2^; Braf^CA/+^; Pten^lox/lox^* mice, histological analysis of *Plp1::CreER^T2^; Braf^CA/+^; Pten^lox/lox^* tumors revealed no signs of pigmentation at the microscopic level (Fig. 2A). One of the few commonalities between MPNSTs and melanomas was the expression of the NC derivative marker Sox10 (Fig. 2B). However, whereas Ngfr was broadly expressed in MPNST, its expression was restricted to few cells in melanoma (Fig. 2B). Conversely, Ctnnb1 expression was prominent in melanoma, but virtually absent in MPNSTs (Fig. 2B). Similarly, the melanocytic markers Tyrp2 (Dct) and Mitf were absent in MPNSTs, whereas they were highly expressed in all melanoma samples (Fig. 2C).

Unlike melanoma, known for their upregulation of and dependency on histone methyltransferase polycomb repressive complex 2 (PRC2) activity (Zingg et al., 2015, 2017, 2018), H3K27Me3 levels remained low in MPNST cells, as supported by clinical evidence in certain patient subsets (Cleven et al., 2016; Prieto-Granada et al., 2016) and other established MPNST models (Brockman et al., 2022; Brosseau et al., 2018) (Fig. 2D). Similarly, compared to melanoma, strongly reduced expression of the PRC2 subunit Ezh2 was detected in MPNST, both in tumor cells and in the surrounding stroma (Fig. 2D). Notably, no substantial increase in the activating histone mark H3K27Ac was observed when compared with *Tyr::CreER^T2^-*driven melanomas (Fig. 2E). The immunohistological characteristics of the latter were consistent with observations in other murine melanoma models, such as *Tyr::CreER^T2^; tdTomato^lox^; Tyr::Nras^Q61K^; Cdkn2a^−/−^*-derived skin tumors (Shakhova et al., 2012; Zingg et al., 2018) (Fig. S1A-F).

### Braf^V600E^ *Pten* haplo-insufficiency variant and initiation compartment

**Fig. 3.**
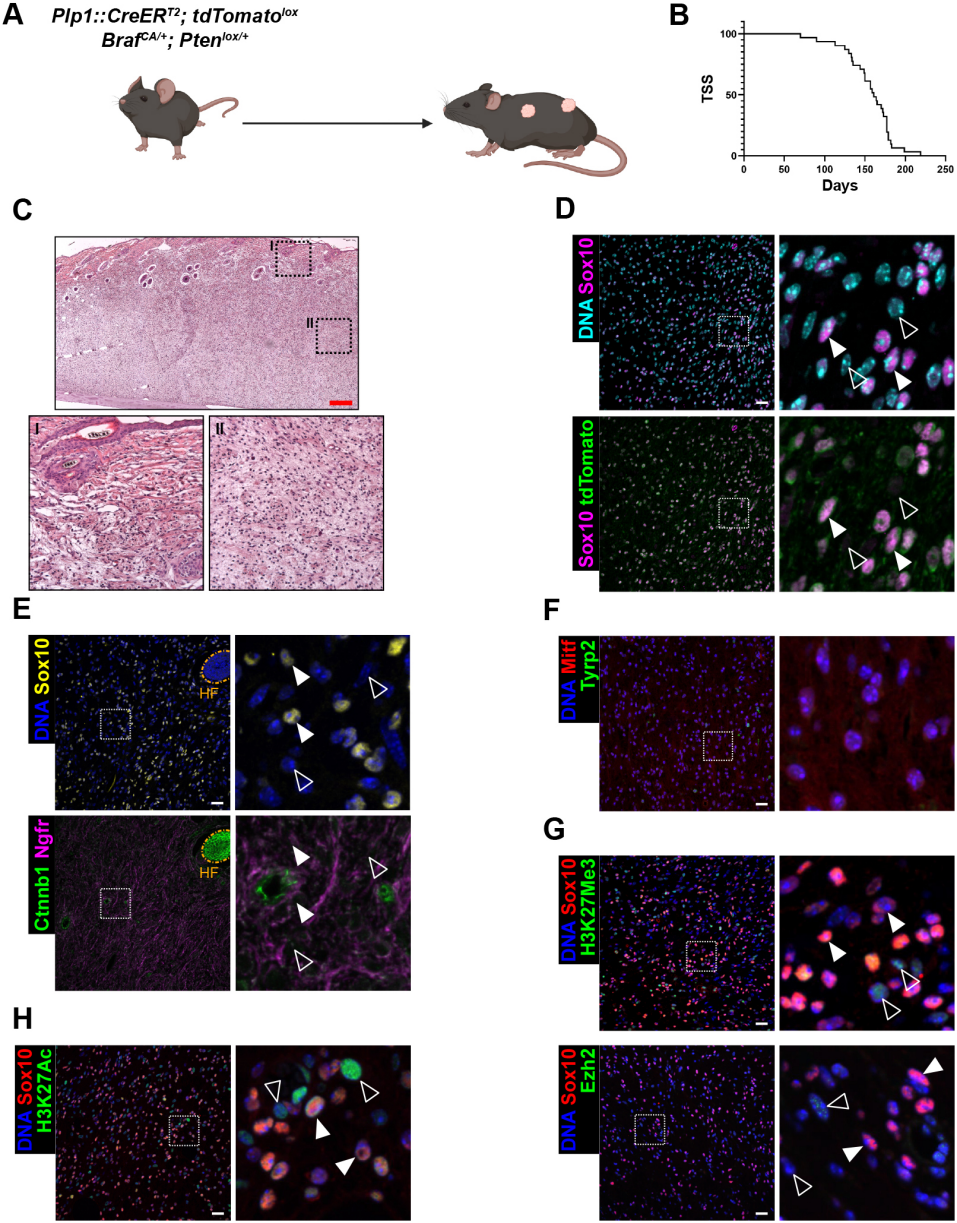
***Pten* haplo-insufficiency recapitulates *Pten* homozygous cKO model with a delayed onset.** (A) Allelic composition of *Pten* haplo-insufficient MPNST model. (B) Kaplan–Meier curve representing the tumor-specific survival (TSS) of *Plp1::CreER^T2^*; *Braf^CA/+^*; *Pten^lox/+^* animals (median survival=161 days; *n*=31 mice) without TM induction. (C-H) Histology of spontaneous *Plp1::CreER^T2^*; *Braf^CA/+^*; *Pten^lox/+^* skin tumors with overview and high-magnification views of the boxed areas. (C) Hematoxylin-Eosin staining with detailed views of epidermal region (I) and deeper dermal region (II). Scale bar: 250 µm. (D-H) IF staining for Sox10 and tdTomato (D), Sox10, Ctnnb1 and Ngfr (E), Mitf and Tyrp2 (F), Sox10, H3K27Me3 and Ezh2 (G), and Sox10 and H3K27Ac (H). (D-H) DNA counterstained with Hoechst 33342. Orange dashed lines label the HF. White arrowheads indicate tumor cells; unfilled arrowheads, stromal cells. Images are representative of *n*>10 (A−F), *n*=3 (G−H) samples. Scale bars: 25 µm.

Owing to the short latency of tumor initiation and its multifocal aspect in *Plp1::CreER^T2^; Braf^CA/+^; Pten^lox/lox^* mice, we turned to a less-severe genotype heterozygous for *Pten^lox^* (Fig. 3A). With delayed tumor onset (Fig. 3B), these mice recapitulated the tumor phenotype observed in their homozygous counterparts. Histologically, tumors from *Plp1::CreER^T2^; Braf^CA/+^; Pten^lox/+^* animals were indistinguishable from their *Pten^lox/lox^* counterparts (Fig. 3C-H), including the extensive infiltration of non-traced (tdTomato^−^ and/or Sox10^−^) stromal cells, which was markedly higher compared to melanoma samples (Fig. 3D, Fig. S1B).

**Fig. 4.**
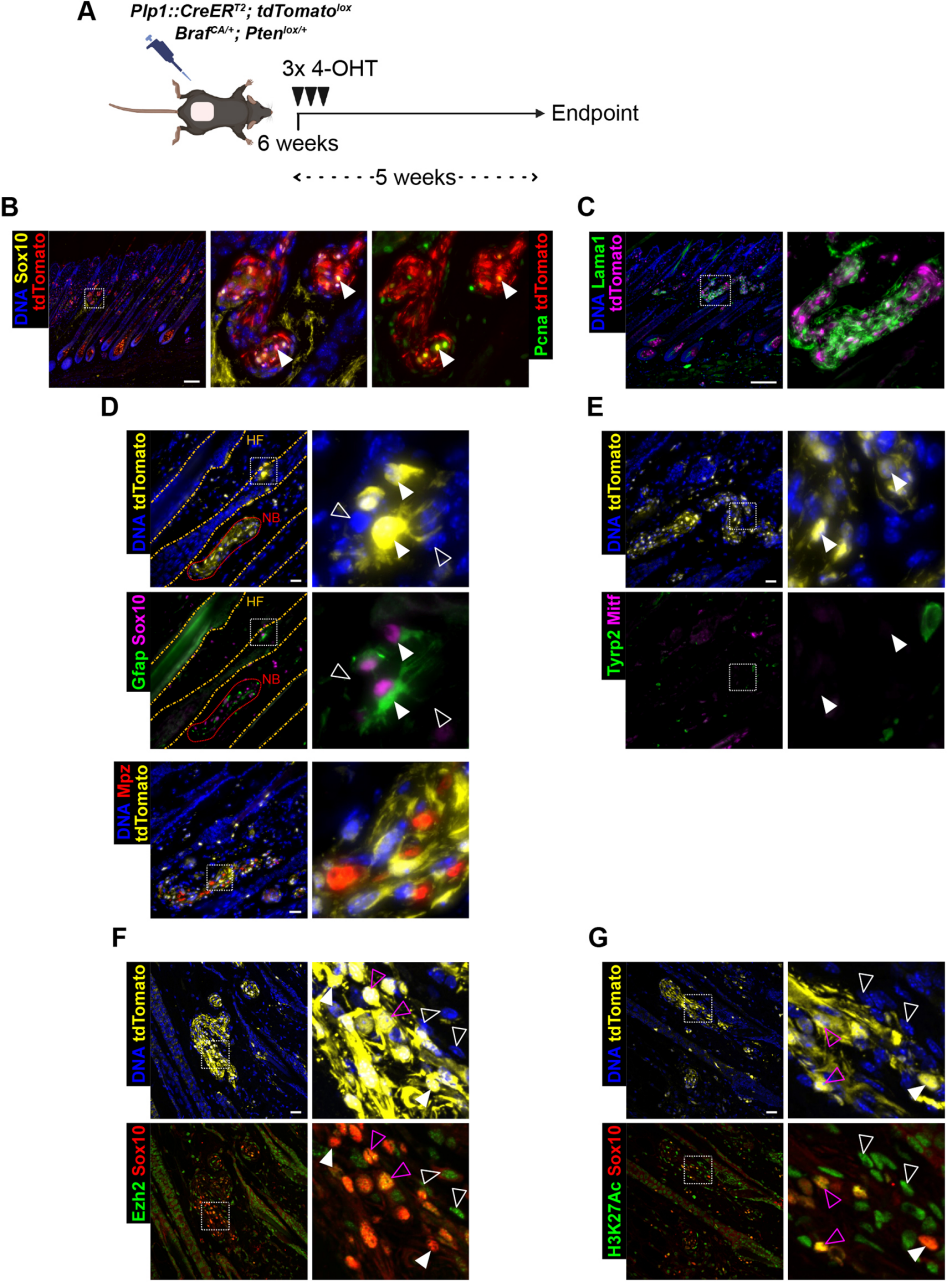
**4-OHT-mediated induction provides molecular clues in compartment of origin.** (A) Schematics of the topical induction strategy to study initiating events in this MPNST model. The back skin of 6-week-old transgenic mice was topically treated with 4-OHT three times and collected after 5 weeks. (B-G) IF staining of TM-treated *Plp1::CreER^T2^*; *Braf^CA/+^*; *Pten^lox/+^* skin samples with a focus on hypertrophic nerve-related structures for Sox10 and the proliferation marker Pcna (B), Lama1 (C), the glial markers Gfap and Mpz (D), the melanocytic markers Mitf and Tyrp2 (E), and the epigenetic markers Ezh2 (F) and H3K27Ac (G). Genetically recombined cells are labeled with tdTomato, and DNA with Hoechst 33342. White arrowheads indicate tdTomato-traced glial cells; unfilled arrowheads, untraced stromal cells; purple arrowheads, triple-positive glial cells (F,G). Orange dashed lines label HFs, red dotted lines label nerve bundles (NBs). Images are representative of *n*=5 samples. Scale bars: 150 µm (B,C); 25 µm (D-G).

The extended tumor-free period in *Pten^lox/+^* mice allowed us to investigate the cellular compartment of origin of these skin tumors using the initially intended TM-induction approach. However, systemic TM treatment in *Plp1::CreER^T2^; Braf^CA/+^* mice was not viable beyond 5 days post-treatment. To circumvent this, we applied repeated topical 4-hydroxy-tamoxifen (4-OHT) treatments to a trimmed patch of back skin (Fig. 4A). Five weeks post-4-OHT induction, skin samples from *Plp1::CreER^T2^; tdTomato^lox^; Braf^CA/+^; Pten^lox/+^* mice showed extensive hypertrophy of tdTomato^+^ nerves located around HFs as well as recombination of melanocytes confined in the bulbs of anagen HFs. The hypertrophic structures expressed the proliferation marker Pcna and the glial markers Sox10, Gfap and Mpz, were embedded in the nerve matrix marker laminin (Lama1) and were negative for the melanocytic markers Tyrp2 and Mitf (Fig. 4B-E). Furthermore, tdTomato-traced cells within hypertrophic nerves displayed notable heterogeneity in Ezh2 expression, consistent with the idea that cells in premalignant lesions start to lose this marker (Fig. 4F). Likewise, the activating histone mark H3K27Ac was expressed at variable levels (Fig. 4G). As previously reported, *Plp1::CreER^T2^* shows recombination capacities in at least a subset of skin melanocytes (Debbache et al., 2018; Hari et al., 2012). However, tdTomato-traced melanocytes in hair bulbs retained unambiguous expression of these melanocytic markers, with no sign of dissemination outside the HF compartment (Fig. S2C-E). These histological and immunohistochemical data are consistent with a focal expansion of the Schwann cell lineage upon recombination, and present glia as a possible origin of the skin tumors observed at later time points.

### MPNSTs from *Braf^CA^; Pten* cKO sciatic nerve allografts are resistant to expression of melanoma markers

**Fig. 5.**
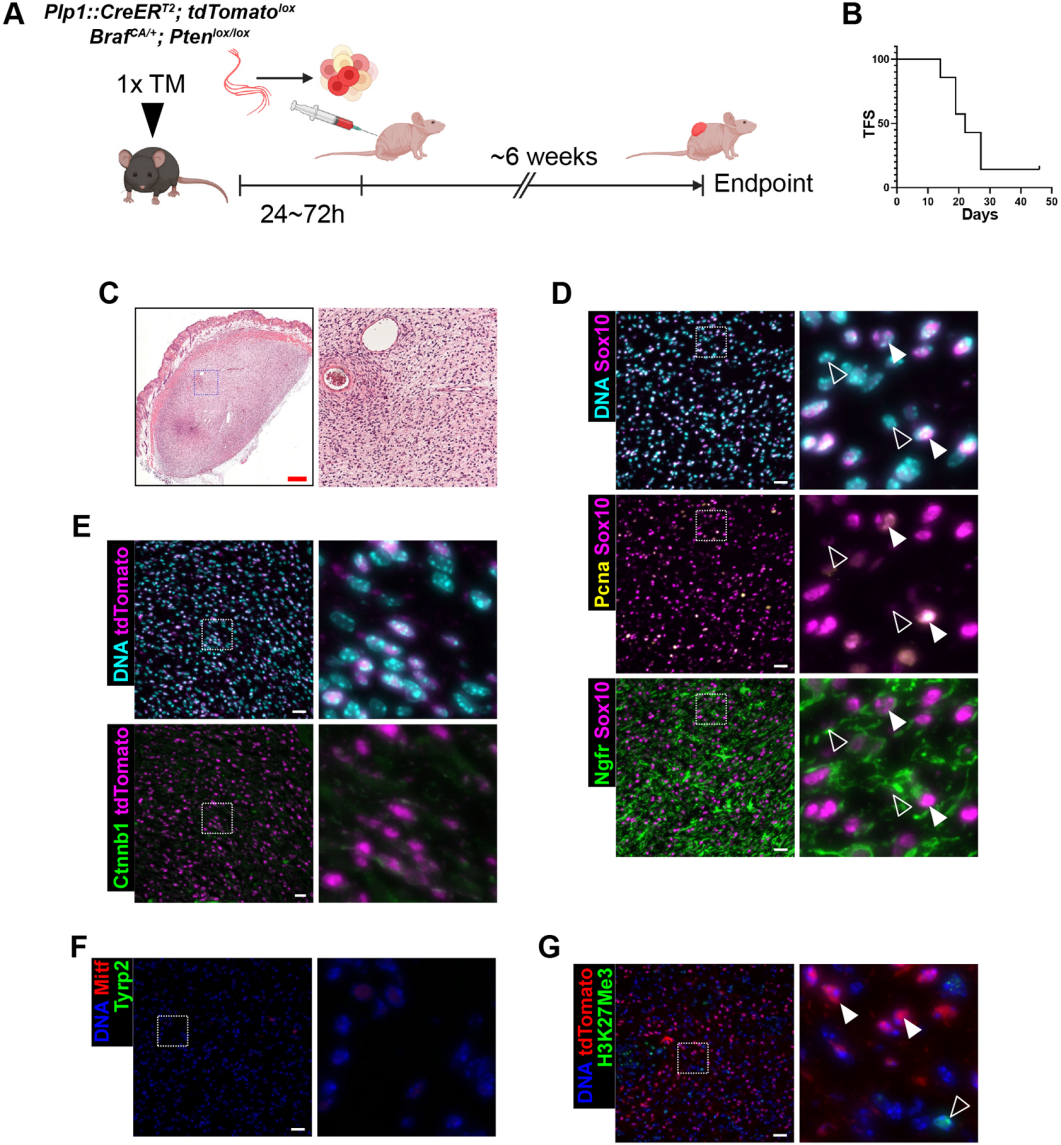
**Transplantation model from sciatic nerve-derived cells shows histological features of MPNSTs.** (A) Schematics of the transplantation model from *Plp1::CreER^T2^*; *Braf^CA/+^*; *Pten^lox/lox^* sciatic nerve cells following *in vivo* TM induction. (B) Kaplan–Meier curve representing the tumor free survival (TFS) of athymic *Foxn1* KO recipients (median latency=22 days; *n*=7 mice). (C-G) Histology of skin allograft tumors with overview (left) and high-magnification view of the boxed area (right). (C) Hematoxylin-Eosin staining. (D-G) IF staining for Sox10, Pcna and Ngfr (D), Ctnnb1 and tdTomato (E), the melanocytic markers Mitf and Tyrp2 (F), and tdTomato and H3K27Me3 (G). (D-G) DNA counterstained with Hoechst 33342. Arrowheads indicate tumor cells, unfilled arrowheads stromal cells. Scale bars: 500 µm (C); 25 µm (D-G).

To rule out potential contamination of the melanocytic compartment, we moved to a different tissue source to further investigate the association of our *Braf^CA^*; *Pten* conditional knockout (cKO)-driven MPNST model with peripheral nerves. After a short systemic TM induction, the sciatic nerves of *Plp1::CreER^T2^; Braf^CA/+^; Pten^lox/lox^* were dissociated and transplanted into partly immunocompromised Nude-*Foxn1^nu/nu^* mice (Fig. 5A). In less than 30 days, most of the animals developed visible subcutaneous tumors with histological features comparable to the skin tumors observed in the spontaneous transgenic model at the end point (Fig. 5B-G).

**Fig. 6.**
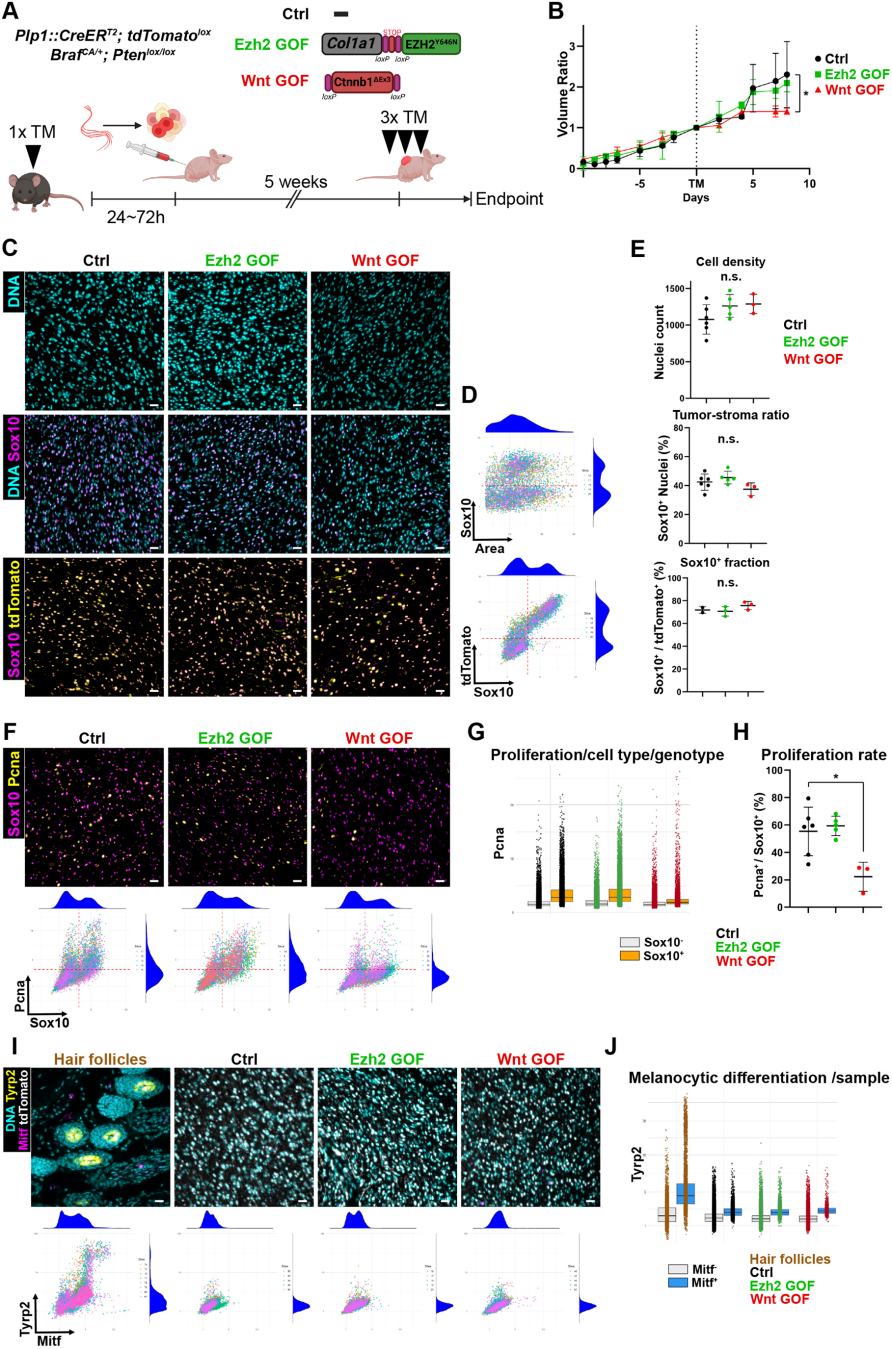
**Activation of Wnt signaling and PRC2 activity fail to promote melanoma features in MPNST.** (A) Schematics of the transplantation model from *Plp1::CreER^T2^*; *Braf^CA/+^*; *Pten^lox/lox^* sciatic nerve cells following *in vivo* TM induction and strategy to conditionally overexpress Ezh2^Y646N^ (Ezh2 GOF) or deplete *Ctnnb1* Exon 3 (Wnt GOF). (B) Tumor growth combined per genotype and normalized to the time of host TM induction. *Plp1::CreER^T2^*; *Braf^CA/+^*; *Pten^lox/lox^* (Ctrl) *n*=6; *Plp1::CreER^T2^*; *Braf^CA/+^*; *Pten^lox/lox^*; *Ezh2^Y646N/+^* (Ezh2 GOF) *n*=5; *Plp1::CreER^T2^*; *Braf^CA/+^*; *Pten^lox/lox^*; *Ctnnb1^Ex3-lox/+^* (Wnt GOF) *n*=3. Error bars represent ±standard deviation. (C) Representative IF images of TM-induced MPNST samples depicting cell density, proportion of Sox10 expressing (Sox10^+^) tumor cells to stromal cells, and proportion of Sox10^+^ tdTomato-traced tumor cells. (D) IF data representation of segmented nuclei for a single biological sample. The upper plot shows normalized Sox10 intensities per area per nucleus per image (*n*=5 per sample) with density distributions on the plot margins. The lower plot displays normalized tdTomato intensities per Sox10 per nucleus per image, also with aggregated density distribution on the margins for the same sample. (E) IF data quantification related to segmented images and grouped per genotypes. Top: Averaged segmented nuclei count per image for each biological sample (Ctrl *n*=6; Ezh2 GOF *n*=5; Wnt GOF *n*=3). Middle: Average proportion of Sox10^+^ (normalized intensity >2) segmented nuclei per image for each biological sample (Ctrl *n*=6; Ezh2 GOF *n*=5; Wnt GOF *n*=3). Bottom: Average proportion of Sox10^+^ (normalized intensity >2) segmented tdTomato^+^ (normalized intensity >2) nuclei per image for each biological sample (tdTomato Ctrl *n*=2; tdTomato Ezh2 GOF *n*=2; tdTomato Wnt GOF *n*=3). (F) Representative IF images per genotype depicting cycling cells (Pcna) and the tumor marker Sox10 with their respective representation of segmented IF data. (G) Normalized Pcna intensities per cell aggregated per Sox10 expression level (<2: stroma; ≥2: tumor) and genotype (Ctrl *n*=6; Ezh2 GOF *n*=5; Wnt GOF *n*=3). (H) Average proportion of Pcna^+^ (normalized intensity >2) segmented Sox10^+^ (normalized intensity >2) nuclei per image for each biological sample (Ctrl *n*=6; Ezh2 GOF *n*=5; Wnt GOF *n*=3). Error bars represent ±standard deviation. (I) Representative IF images of host hair follicles and MPNST samples per genotype depicting Tyrp2 and Mitf melanocytic markers as well as tumor marker tdTomato with their respective representation of segmented IF data. (J) Normalized Tyrp2 intensities per cell aggregated per Mitf expression level (<2 or ≥2) and genotype (Ctrl *n*=6; Ezh2 GOF *n*=5; Wnt GOF *n*=3). In G and J, box limits represent the interquartile range between the 25th and 75th percentile. and horizontal line within box the median. Scale bars: 25 µm (C,F,I). *P<0.05 (unpaired nonparametric Mann–Whitney rank test). n.s., not significant (*P*>0.05).

The MPNST and melanoma models in our study are genetically identical, but with different, although developmentally related, cells of origin. Therefore, we aimed to examine separately the effect of two well-established melanoma drivers on MPNST cells: PRC2 activity (‘Ezh2 GOF’) and canonical Wnt signaling (‘Wnt GOF’), with the aim to specifically assess the transdifferentiation/plasticity potential of this tumor type. Ezh2 GOF was enacted using the *ColA1-LSL-Ezh2^Y646N^* allele (Béguelin et al., 2013), which allows conditional Ezh2^Y646N^ expression upon Cre recombination and suffices for melanoma initiation upon MAPK activation (Zingg et al., 2018). Wnt GOF was achieved using the *Ctnnb1^Ex3-lox^* allele, which allows *Ctnnb1* exon 3 depletion and subsequent Ctnnb1 stabilization upon Cre recombination and promotes melanoma initiation in the same oncogenic setting (Damsky et al., 2011) (Fig. 6A). To this end, sciatic nerves of *Plp1::CreER^T2^; Braf^CA/+^; Pten^lox/lox^* animals bearing the additional alleles of interest were harvested after a short TM induction, dissociated and transplanted in partly immunocompromised Nude-*Foxn1^nu/nu^* mice as described above. After an initial growth phase of about 5 weeks, Nude-*Foxn1^nu/nu^* hosts were further treated with TM to assure efficient recombination of target genes, upon which tumor growth of the Wnt GOF group was decreased, while tumors of Ctrl and Ezh2 GOF groups remained unaffected (Fig. 6B).

Histological and segmentation analysis of sciatic nerve-derived MPNST samples functionally validated the genetic models used in these experiments. First, we confirmed an increase in PRC2 activity in tumor tissues from Ezh2 GOF mice, as evidenced by elevated H3K27Me3 levels compared to other genotypes. This resulted in a higher proportion of cells surpassing a predefined H3K27Me3^HIGH^ threshold, which was consistently applied across all samples (Fig. S3A-C). In parallel, tdTomato^+^ tumor cells in Wnt GOF samples displayed increased nuclear Ctnnb1 levels relative to tdTomato^−^ stromal cells. This finding corresponded to a greater number of Ctnnb1^HIGH^ cells, based on a uniformly set threshold for all samples (Fig. S3D-F). While no significant differences were observed in cell density or tumor-to-stroma cell ratios across genotypes, the macroscopic reduction in tumor growth observed in Wnt GOF mice was confirmed at the cellular level by a decrease in proliferating (Pcna^+^ Sox10^+^) tumor cells (Fig. 6C-H).

Further, we investigated whether increased PRC2 activity or Wnt signaling in MPNST cells would be sufficient to drive a switch to a melanocytic fate and, ultimately, to melanoma formation from peripheral nerves. However, at least within the time span of the experiment (8 days after TM induction), neither Ezh2 GOF nor Wnt GOF could achieve an increase in melanocytic marker expression in MPNST cells (Fig. 6I,J). These findings suggest that, in this tumorigenic context of Braf^V600E^/*Pten* cKO, the tissue or cellular compartment of origin plays a dominant role in determining tumor phenotype, even when pro-melanoma signaling pathways are activated.

## DISCUSSION

This study introduces a novel murine model of sporadic, *Nf1* wild-type MPNSTs, leveraging the concurrent activation of MAPK and PI3K signaling pathways via Braf^V600E^ expression and conditional knockout of *Pten*, respectively. Although *BRAF^V600E^*-mutated MPNSTs are rarely reported clinically, their true prevalence may be underestimated, as *BRAF* status is often used diagnostically to favor melanoma over MPNST, and some amelanotic melanomas, especially the spizoid subtype, are molecularly challenging to diagnose (Dimonitsas et al., 2018; Garrido-Ruiz et al., 2010). Similarly, although *PTEN* loss is not commonly observed as a primary genetic alteration in patients, PTEN downregulation has been proposed as a key contributor to disease progression, and *Pten* loss has therefore been incorporated into non-inducible murine MPNST models by others (Gregorian et al., 2009; Keng et al., 2012). Hence, the *Plp1::CreER^T2^; Braf^CA^; Pten^lox^* allelic combination successfully established a versatile and robust tumor model with near-complete penetrance and, importantly, short latency. This system enables tumor initiation either spontaneously through localized 4-OHT induction or via systemic TM treatment followed by cell transplantation and would be well suited to study early events in cell transformation and disease progression of a peripheral glia-derived tumor. Importantly, without additional genetic manipulation, the model intrinsically reflected PRC2 complex alterations, a key driving force of MPNST oncogenic biology (Zhang et al., 2022). This suggests that epigenetic regulation may arise as a direct consequence of the activated signaling pathways in this specific cellular context.

Although tumors driven by both *Plp1::CreER^T2^* and *Tyr::CreER^T2^* share the same genetic alterations, they diverged significantly in their phenotypic characteristics during tumor expansion. In our hands, *Plp1::CreER^T2^* efficiently recombined melanocytes (Debbache et al., 2018); however, under the same genetic conditions, tumor formation and/or significant expansion of the melanocytic compartment systematically failed. Instead, hypertrophic structures induced by TM treatment expressed glial markers and were strongly associated with nerves. In parallel, *Tyr::CreER^T2^; Braf^CA/+^; Pten^lox/+^* mice exhibited a median melanoma-free survival of approximately 250 days (Dankort et al., 2009), suggesting that the melanocytic compartment is relatively resistant to tumor initiation in the *Braf^CA/+^; Pten^lox/+^* genetic context. These findings suggest that the cell of origin plays a crucial role in shaping tumor phenotype, beyond the expression of broadly shared NC markers such as Sox10 or S100b. This phenotypic divergence may reflect lineage-specific transcriptional or epigenetic programs that persist long after tumor initiation and influence subsequent progression.

Moreover, the lack of melanocytic expansion in the interfollicular space following Braf^V600E^ induction by *Plp1::CreER^T2^*, contrasting with previous reports using *Tyr::CreER^T2^* (Shakhova et al., 2012), raises the possibility of negative crosstalk between glial and melanocytic lineages. Activated glia have been shown to promote TGFβ signaling in the granulation tissue of skin wounds, contributing to myofibroblast specification (Parfejevs et al., 2018). Because TGFβ is known to inhibit melanocyte proliferation (Nishimura et al., 2010), a glia-dominated microenvironment may suppress melanocytic expansion, particularly in dermal tissues.

To explore lineage dynamics in NC-derived tumors, we investigated the impact of canonical Wnt signaling in peripheral glia-derived MPNSTs. During embryonic development, Wnt signal activation in migratory NC cells promotes melanocyte formation and expansion, but this effect is stage dependent and not observed at an earlier stage of NC development (Hari et al., 2012). Of note, Wnt activation in established MPNSTs failed to promote a shift toward a melanocytic phenotype. Furthermore, in contrast to its pro-proliferative role in melanoma (Damsky et al., 2011; Delmas et al., 2007), activation of canonical Wnt signaling in MPNSTs counteracted tumor cell proliferation. Likewise, *Tyr::CreER^T2^* - driven Ezh2 GOF, which results in canonical Wnt signal activation in the melanocyte lineage, promotes melanoma formation (Zingg et al., 2018), whereas *Plp1::CreER^T2^* -driven Ezh2 GOF in MPNSTs had no overt effect on the tumor phenotype. It is well established that developmental programs reminiscent of embryonic NC become reactivated in melanoma (Diener et al., 2021), and it is conceivable that this also occurs in our MPNST model, similar to processes described in MPNSTs driven by genetic loss of *NF1* and PRC2 deficiency (Zhang et al., 2022). However, given their distinct responsiveness to melanoma-driving cues, the cellular states characteristic for melanoma and MPNST, respectively, appear to be distinct, at least in our mouse models. It will be interesting to test this hypothesis, for instance by in-depth analysis of cell compositions at the single-cell level in murine and human tumor samples.

In summary, although our study is limited by the depth of molecular analyses and the absence of additional genetic alterations most commonly observed in MPNSTs, such as *Cdkn2a* or *Tp53* loss, it provides a robust foundation for further refinement of sporadic non-*NF1* based MPNST modeling. This system not only improves latency and penetrance compared with previously published models but also recapitulates a broader spectrum of MPNSTs with its intrinsic loss of PRC2 activity and high stromal infiltration. Further, it emphasizes the need to consider both genetic context and cellular origin in interpreting tumor behavior, both in preclinical studies and in the clinic. Future studies integrating robust lineage tracing, epigenomic profiling, and longitudinal imaging may help clarify the mechanisms underlying the observed phenotypes and improve the translational utility of this preclinical MPNST model.

## MATERIALS AND METHODS

### Mice

Female Hsd:Athymic Nude-*Foxn1^nu/nu^* mice (8-10 weeks old) were purchased (Envigo). The mouse strain carrying the *Plp1::CreER^T2^* allele (MGI:2663093) was obtained from Ueli Suter's laboratory (ETH Zurich, Switzerland). Mouse strains carrying *tdTomato^LSL^* (MGI:3809524), *Braf^CA^* allele (MGI:3711771), *Pten^lox^* allele (MGI:2156086) and *Ctnnb1^Ex3-lox^* (MGI:1858008) were imported from The Jackson Laboratory. The mouse strain carrying a Cre-inducible *ColA1-LSL-Ezh2^Y646N^* allele (MGI:5519911) was a gift from Kwok-Kin Wong (Dana-Farber Cancer Institute, USA). MPNST models were obtained by serial intercrosses of single transgenic lines to achieve mouse genotypes as indicated in Figs 1A, 2A, 3A,H, 4A and 5A. Experimental animals were maintained on a predominantly C57BL/6J background, although the exact genetic composition was not determined. Litters were obtained at expected Mendelian ratios, and no sex-related differences in tumor incidence were observed.

No statistical methods were used to predetermine sample size. The experiments were not randomized and the investigators were aware of group allocation during experiments and outcome assessment. The mouse colony was housed in certified animal facilities with a 12-h light/dark cycle in a temperature-controlled room (22±1°C) with free access to water and food, in accordance with Swiss guidelines. Animal experiments were performed under approved permits reviewed by the veterinary authorities of Canton of Zurich, Switzerland, and were performed in accordance with Swiss animal welfare laws.

### Breeding schemes

#### MPNST *Pten* cKO

Experimental animals were obtained by crossing two separate colonies of *Plp1::CreER^T2^* ; *Pten^lox/lox^* mice with *tdTomato^lox/lox^*; *Braf^CA/CA^; Pten^lox/lox^* mice due to the tumor incidence of the model reaching 100% penetrance without TM treatment.

#### MPNST *Pten* haplo-insufficient

Experimental animals were obtained by crossing two separate colonies of *Plp1::CreER^T2^* mice with *tdTomato^lox/lox^*; *Braf^CA/CA^; Pten^lox/lox^*. *Plp1::CreER^T2^* transgenes always kept heterozygous.

#### Ezh2 GOF and Wnt GOF strains

The *ColA1-LSL-Ezh2^Y646N^* and *Ctnnb1^Ex3-lox^* alleles were introduced in separate substrains of *tdTomato^lox/+^*; *Braf^CA/CA^; Pten^lox/lox^* bearing mice, which were subsequently bred with *Plp1::CreER^T2^*; *Pten^lox/lox^* animals to obtain the desired genotypes.

### Mouse genotyping

Mouse toe or ear biopsies were processed using the MyTaq Extract-PCR kit (Labgene, BIO21127) in a single-tube format. Each sample was lysed in 100 µl of buffer for 10 min at 75°C with shaking followed by inactivation for 10 min at 95°C. Tissue debris was separated from DNA containing supernatant by centrifugation at 13,000 ***g*** for 1 min. For each 10 µl PCR reaction, 2 µl of DNA supernatant was used with MyTaq HS Red Mix and a routine program with a 60°C annealing temperature and 36 cycles for all genes of interest. Primers are listed in Table S1.

PCR products were segregated using a 6 V cm^−1^ electric field for 35 min in 1× TAE with 2.5% agarose gel, counterstained with Red Safe (iNtRON Biotechnology, 21141).

### Tissue blocks from melanoma models

Melanoma samples from *Tyr::CreER^T2^; Braf^CA/+^; Pten^lox/lox^* and *Tyr::CreER^T2^; tdTomato^LSL^; Tyr::NrasQ61K; Ink4a^−/−^* mice used in comparative histology panels were obtained from a cohort of samples described by Zingg et al. (2018).

### Local gene activation and deletion

Under sustained isoflurane anesthesia, 6-week-old transgenic animals were topically treated with 20 µl of 4-OHT (Sigma-Aldrich, H7904) 4 mg ml^−1^ in DMSO-ethanol solution (1:4) onto 2 cm^2^ of trimmed back skin daily over three days.

### Sciatic nerve cell transplantation

To isolate recombined sciatic nerve cells from MPNST *Pten* cKO control, Ezh2 GOF and Wnt GOF animals, a single 200 µl injection of 10 mg ml^−1^ TM was performed intra-peritoneally. Femur-length sciatic nerves segments were harvested 24-72 h post recombination, dissected into small pieces and the tissue was digested using 0.25 mg/ml Liberase DH Research Grade (Roche, 05401054001) in RPMI 1640 for 45 min at 37°C with gentle rocking followed by a treatment with 0.2 mg ml^−1^ DNase I (Roche, 10104159001) for an additional 15 min. Cell clumps were further separated using 70 µm pore size cell strainers (VWR, 734-2761). Cells were pelleted by centrifugation at 300 ***g*** for 5 min and resuspended in 100 µl RPMI 1640 complemented with 10% fetal bovine serum (Thermo Fisher Scientific, 16140) and Penicillin-Streptomycin (Thermo Fisher Scientific, 15070), then 50 µl of the cell suspension was injected subcutaneously in the lower back region of Athymic Nude hosts under sustained isoflurane anesthesia.

### Murine tissue samples

Mouse skin and tumor samples were fixed in 4% Roti-Histofix (Carl Roth, P087.3) overnight at 4°C shortly after euthanasia, embedded in paraffin, and sliced into 5 µm sections. Sections were deparaffinized in UltraClear (VWR, 3905.5000PE) followed by an ethanol series of decreasing concentration. Rehydrated sections were subjected to antigen unmasking in 0.2× citrate buffer (Agilent, S2369) using a vegetable steamer for 20 min and cooled back to room temperature for 10 min in a water bath before further processing. Sections were then permeabilized for 5 min in PBS and 0.2% Triton X-100 (Sigma-Aldrich, T8787) and blocked with 3% donkey serum (VWR, S2170-050) in PBS and 0.05% Triton X-100 (PBST) for 1 h. An additional step of Mouse-on-Mouse IgG Blocking solution (Thermo Fisher Scientific, R37621, 1:30) was used for 30 min, when applicable. Primary antibodies against CD45 (BD Biosciences, 550539, RRID:AB_2174426, 1:200), Ctnnb1 (Santa Cruz Biotechnology, sc-7199, RRID:AB_634603, 1:300), Ezh2 (Cell Signaling Technology, 5246, RRID:AB_10694683, 1:200), Gfap (Agilent, Z0334, RRID:AB_10013382, 1:500), H3K27Ac (Cell Signaling Technology, 8173, RRID:AB_10949503, 1:200) H3K27Me3 (Cell Signaling Technology, 9733, RRID:AB_2616029, 1:2000), Lama1 (Sigma-Aldrich, L9393, RRID:AB_477163, 1:250), mast cell chymase (Proteintech, 18189-1-AP, RRID:AB_2083611, 1:200), Mitf [mouse monoclonal 6D3, gift from Heinz Arnheiter's lab, Scientist emeritus, National Institute of Neurological Disorders and Stroke (NINDS)/NIH, Bethesda, MD, USA, 1:200], Mpz (Thermo Fisher Scientific, PA5-37179, RRID:AB_2553943, 1:100), Ngfr (goat polyclonal: R&D Systems, AF1157, RRID:AB_2298561, 1:100; rabbit polyclonal: Alomone Labs, ANT-007, RRID:AB_2039968, 1:200), Pcna (Thermo Fisher Scientific, 13-3900, RRID:AB_2533016, 1:200), Sox10 (custom-made rabbit polyclonal raised against hSOX10 50-65 ELGKVKKEQQDGEADD, 1:2000; mouse monoclonal: Proteintech, 66786-1-Ig, RRID:AB_2882131, 1:500), tdTomato [LSBio (LifeSpan), LS-C340696, RRID:AB_2819022, 1:500] and Tyrp2 (custom-made rabbit polyclonal raised against hTYRP2 67-84 DTRPWSGPYILRNQDDRE, 1:5000) were applied in PBST overnight at 4°C. Slides were washed with PBST three times for 5 min each wash. Primary antibodies were visualized using Alexa Fluor (AF) 488 anti-rabbit (Jackson ImmunoResearch, 711-545-152, RRID:AB_2313584, 1:500), AF 488 anti-mouse (Jackson ImmunoResearch, 715-545-150, RRID:AB_2340846, 1:500), Cy3 anti-goat (Jackson ImmunoResearch, 705-165-147, RRID:AB_2307351, 1:500), Cy3 anti-mouse (Jackson ImmunoResearch, 705-165-147, RRID:AB_2307351, 1:500), Cy3 anti-rabbit (Jackson ImmunoResearch, 711-165-152, RRID:AB_2307443l, 1:500), AF 647 anti-goat (Jackson ImmunoResearch, 705-605-147, RRID:AB_2340437, 1:500), AF 647 anti-rabbit (Jackson ImmunoResearch, 711-605-152, RRID:AB_2492288, 1:500), secondary antibodies in PBST together with 1 µg ml^−1^ Hoechst 33342 (Sigma-Aldrich, 14533) for DNA counterstain for 1 h at room temperature. After another series of three washes in PBST each for 5 min and slides were mounted with ProLong™ Gold Antifade Mountant (Thermo Fisher Scientific, P36930) and #1.5 glass cover slips (CellPath, SAH-2450-03A).

### Hematoxylin-Eosin-stained tissue sections

H&E-stained sections of murine MPNSTs were independently reviewed by a board-certified neuropathologist at the Department of Neuropathology, University Hospital Zurich. The assessment encompassed key histopathological parameters, including cellular density and morphology, extracellular matrix composition, and the extent and nature of immune infiltration.

### Immunofluorescence images

Single or tile-scanned maximum intensity projected (MIP) multi-channel 7 µm stack images were acquired using an Olympus IXplore SpinSR10 equipped with a Yokogawa CSU-W1 50 μm pinhole disk, UPLAN S Apo 40×/NA 0.95 air objective. Dual excitation of Hoechst 33342/Cy3 and AF 488/AF 647 was sequentially performed using 405 nm and 561 nm, followed by 488 nm and 640 nm lasers. Dual emission was detected using an image splitting 561 nm long-pass (LP) dichroic mirror, with images being captured by two Hamamatsu ORCA-Fusion sCMOS cameras (2304×2304 pixels, 6.5×6.5 µm pixel size). Band pass filters employed included 447/60 and 617/73, followed by 525/50 and 685/40 for emission channels Hoechst 33342/Cy3 and AF 488/AF 647 (Figs 1G,H, 2B-E, 3D-H, 4F,G, Fig. S1B-F) or a Leica DMI6000 widefield microscope with an HCX PL Apo 40×/NA 1.25 oil immersion objective (all other fluorescence images). In this case, sequential single-channel acquisition was performed using a white light source in combination with excitation (EX) 355/56 and emission (EM) 460/50 filters for Hoechst 33342, EX 470/40 and EM 525/50 for AF 488, EX 546/10 and EM 585/40 filters for Cy3, and EX 620/60 and EM 700/76 for AF 647 fluorophores, detected with a Leica K8 sCMOS camera (2048×2048 pixels, 6.5×6.5 µm pixel size) and stored as 16-bit image files.

### Semi-automated image quantification

For each MIP, individual cells were identified and segmented on the nuclei counterstain channel using Ilastik (Berg et al., 2019). Segmentation probability masks were binarized using Otsu's thresholding, further segmented using the built-in ‘Watershed’ plugin and stored as image sequences using ImageJ Fiji (Schindelin et al., 2012). The size (area) of each object, file of origin and their respective mean fluorescence intensities obtained from the 16-bit images, acquired in parallel for each channel, were then compiled. Filtered ‘cells’ were defined with nuclear areas ranging from 5 to 60 µm^2^. For each channel, ‘raw’ mean fluorescence intensities were then normalized per MIP to a baseline of ‘negative’ cells set at the mean intensity of lowest 10% of the size-filtered nuclei for that image. Cells were then categorized for each marker based on their normalized intensity above or under a pre-defined threshold identical across all images and samples.

## Supplementary Material

10.1242/dmm.052471_sup1 Supplementary information

## References

[DMM052471C1] Béguelin, W., Popovic, R., Teater, M., Jiang, Y., Bunting, K. L., Rosen, M., Shen, H., Yang, S. N., Wang, L., Ezponda, T. et al. (2013). EZH2 is required for germinal center formation and somatic EZH2 mutations promote lymphoid transformation. *Cancer Cell* 23, 677-692. 10.1016/j.ccr.2013.04.011 23680150 PMC3681809

[DMM052471C2] Berg, S., Kutra, D., Kroeger, T., Straehle, C. N., Kausler, B. X., Haubold, C., Schiegg, M., Ales, J., Beier, T., Rudy, M. et al. (2019). ilastik: interactive machine learning for (bio)image analysis. *Nat. Methods* 16, 1226-1232. 10.1038/s41592-019-0582-9 31570887

[DMM052471C3] Brockman, Q. R., Scherer, A., McGivney, G. R., Gutierrez, W. R., Voigt, A. P., Isaacson, A. L., Laverty, E. A., Roughton, G., Knepper-Adrian, V., Darbro, B. et al. (2022). PRC2 loss drives MPNST metastasis and matrix remodeling. *JCI Insight* 7, e157502. 10.1172/jci.insight.157502 36066973 PMC9714789

[DMM052471C4] Brosseau, J.-P., Liao, C.-P., Wang, Y., Ramani, V., Vandergriff, T., Lee, M., Patel, A., Ariizumi, K. and Le, L. Q. (2018). NF1 heterozygosity fosters de novo tumorigenesis but impairs malignant transformation. *Nat. Commun.* 9, 5014. 10.1038/s41467-018-07452-y 30479396 PMC6258697

[DMM052471C5] Cleven, A. H., Al Sannaa, G. A., Briaire-de Bruijn, I., Ingram, D. R., van de Rijn, M., Rubin, B. P., de Vries, M. W., Watson, K. L., Torres, K. E., Wang, W.-L. et al. (2016). Loss of H3K27 tri-methylation is a diagnostic marker for malignant peripheral nerve sheath tumors and an indicator for an inferior survival. *Mod. Pathol.* 29, 1113. 10.1038/modpathol.2016.103 27573709

[DMM052471C6] Colombo, S., Petit, V., Wagner, R. Y., Champeval, D., Yajima, I., Gesbert, F., Aktary, Z., Davidson, I., Delmas, V. and Larue, L. (2022). Stabilization of β-catenin promotes melanocyte specification at the expense of the Schwann cell lineage. *Development* 149, dev194407. 10.1242/dev.194407 34878101 PMC8917410

[DMM052471C7] Cortes-Ciriano, I., Steele, C. D., Piculell, K., Al-Ibraheemi, A., Eulo, V., Bui, M. M., Chatzipli, A., Dickson, B. C., Borcherding, D. C., Feber, A. et al. (2023). Genomic patterns of malignant peripheral nerve sheath tumor (MPNST) evolution correlate with clinical outcome and are detectable in cell-free DNA. *Cancer Discov.* 13, 654-671. 10.1158/2159-8290.CD-22-0786 36598417 PMC9983734

[DMM052471C8] Damsky, W. E., Curley, D. P., Santhanakrishnan, M., Rosenbaum, L. E., Platt, J. T., Gould Rothberg, B. E., Taketo, M. M., Dankort, D., Rimm, D. L., McMahon, M. et al. (2011). β-catenin signaling controls metastasis in Braf-activated Pten-deficient melanomas. *Cancer Cell* 20, 741-754. 10.1016/j.ccr.2011.10.030 22172720 PMC3241928

[DMM052471C9] Dankort, D., Curley, D. P., Cartlidge, R. A., Nelson, B., Karnezis, A. N., Damsky, W. E., You, M. J., DePinho, R. A., McMahon, M. and Bosenberg, M. (2009). Braf(V600E) cooperates with Pten loss to induce metastatic melanoma. *Nat. Genet.* 41, 544-552. 10.1038/ng.356 19282848 PMC2705918

[DMM052471C10] Debbache, J., Parfejevs, V. and Sommer, L. (2018). Cre-driver lines used for genetic fate mapping of neural crest cells in the mouse: an overview. *Genesis* 56, e23105. 10.1002/dvg.23105 29673028 PMC6099459

[DMM052471C11] Delmas, V., Beermann, F., Martinozzi, S., Carreira, S., Ackermann, J., Kumasaka, M., Denat, L., Goodall, J., Luciani, F., Viros, A. et al. (2007). Beta-catenin induces immortalization of melanocytes by suppressing p16INK4a expression and cooperates with N-Ras in melanoma development. *Genes Dev.* 21, 2923-2935. 10.1101/gad.450107 18006687 PMC2049194

[DMM052471C12] Dhomen, N., Da Rocha Dias, S., Hayward, R., Ogilvie, L., Hedley, D., Delmas, V., McCarthy, A., Henderson, D., Springer, C. J., Pritchard, C. et al. (2010). Inducible expression of (V600E) Braf using tyrosinase-driven Cre recombinase results in embryonic lethality. *Pigment Cell Melanoma Res.* 23, 112-120. 10.1111/j.1755-148X.2009.00662.x 20002887

[DMM052471C13] Diener, J., Baggiolini, A., Pernebrink, M., Dalcher, D., Lerra, L., Cheng, P. F., Varum, S., Häusel, J., Stierli, S., Treier, M. et al. (2021). Epigenetic control of melanoma cell invasiveness by the stem cell factor SALL4. *Nat. Commun.* 12, 5056. 10.1038/s41467-021-25326-8 34417458 PMC8379183

[DMM052471C14] Dimonitsas, E., Liakea, A., Sakellariou, S., Thymara, I., Giannopoulos, A., Stratigos, A., Soura, E., Saetta, A. and Korkolopoulou, P. (2018). An update on molecular alterations in melanocytic tumors with emphasis on Spitzoid lesions. *Ann. Transl. Med.* 6, 249-249. 10.21037/atm.2018.05.23 30069451 PMC6046302

[DMM052471C15] Garrido-Ruiz, M. C., Requena, L., Ortiz, P., Pérez-Gómez, B., Alonso, S. R. and Rodríguez Peralto, J. L. (2010). The immunohistochemical profile of Spitz nevi and conventional (non-Spitzoid) melanomas: a baseline study. *Mod. Pathol.* 23, 1215-1224. 10.1038/modpathol.2010.102 20543820

[DMM052471C16] Gregorian, C., Nakashima, J., Dry, S. M., Nghiemphu, P. L., Smith, K. B., Ao, Y., Dang, J., Lawson, G., Mellinghoff, I. K., Mischel, P. S. et al. (2009). PTEN dosage is essential for neurofibroma development and malignant transformation. *Proc. Natl. Acad. Sci. USA* 106, 19479-19484. 10.1073/pnas.0910398106 19846776 PMC2765459

[DMM052471C17] Gupta, G., Mammis, A. and Maniker, A. (2008). Malignant peripheral nerve sheath tumors. *Neurosurg. Clin. N. Am.* 19, 533-543. 10.1016/j.nec.2008.07.004 19010279

[DMM052471C18] Hari, L., Miescher, I., Shakhova, O., Suter, U., Chin, L., Taketo, M., Richardson, W. D., Kessaris, N. and Sommer, L. (2012). Temporal control of neural crest lineage generation by Wnt/β-catenin signaling. *Development* 139, 2107-2117. 10.1242/dev.073064 22573620

[DMM052471C19] Hirbe, A. C., Pekmezci, M., Dahiya, S., Apicelli, A. J., Van Tine, B. A., Perry, A. and Gutmann, D. H. (2014). BRAFV600E mutation in sporadic and neurofibromatosis type 1-related malignant peripheral nerve sheath tumors. *Neuro-Oncol.* 16, 466-467. 10.1093/neuonc/not248 24366910 PMC3922521

[DMM052471C20] Kao, E. Y., Wakeman, K. M., Wu, Y., Gross, J. M., Chen, E. Y., Ricciotti, R. W., Liu, Y. J. and Mantilla, J. G. (2022). Prevalence and detection of actionable BRAF V600 and NRAS Q61 mutations in malignant peripheral nerve sheath tumor by droplet digital PCR. *Hum. Pathol.* 129, 90-97. 10.1016/j.humpath.2022.08.005 36067829

[DMM052471C21] Kaplan, H. G., Rostad, S., Ross, J. S., Ali, S. M. and Millis, S. Z. (2018). Genomic profiling in patients with malignant peripheral nerve sheath tumors reveals multiple pathways with targetable mutations. *J. Natl. Compr. Cancer Netw.* 16, 967-974. 10.6004/jnccn.2018.7033 30099373

[DMM052471C22] Kaucka, M., Szarowska, B., Kavkova, M., Kastriti, M. E., Kameneva, P., Schmidt, I., Peskova, L., Joven Araus, A., Simon, A., Kaiser, J. et al. (2021). Nerve-associated Schwann cell precursors contribute extracutaneous melanocytes to the heart, inner ear, supraorbital locations and brain meninges. *Cell. Mol. Life Sci.* 78, 6033-6049. 10.1007/s00018-021-03885-9 34274976 PMC8316242

[DMM052471C23] Keng, V. W., Rahrmann, E. P., Watson, A. L., Tschida, B. R., Moertel, C. L., Jessen, W. J., Rizvi, T. A., Collins, M. H., Ratner, N. and Largaespada, D. A. (2012). PTEN and NF1 inactivation in Schwann cells produces a severe phenotype in the peripheral nervous system that promotes the development and malignant progression of peripheral nerve sheath tumors. *Cancer Res.* 72, 3405-3413. 10.1158/0008-5472.CAN-11-4092 22700876 PMC3428071

[DMM052471C24] Kim, A. and Cohen, M. S. (2016). The discovery of vemurafenib for the treatment of BRAF-mutated metastatic melanoma. *Expert Opin. Drug Discov.* 11, 907-916. 10.1080/17460441.2016.1201057 27327499 PMC5443413

[DMM052471C25] Le, L. Q., Liu, C., Shipman, T., Chen, Z., Suter, U. and Parada, L. F. (2011). Susceptible stages in Schwann cells for NF1-associated plexiform neurofibroma development. *Cancer Res.* 71, 4686-4695. 10.1158/0008-5472.CAN-10-4577 21551250 PMC3145496

[DMM052471C26] Leone, D. P., Genoud, S., Atanasoski, S., Grausenburger, R., Berger, P., Metzger, D., Macklin, W. B., Chambon, P. and Suter, U. (2003). Tamoxifen-inducible glia-specific Cre mice for somatic mutagenesis in oligodendrocytes and Schwann cells. *Mol. Cell. Neurosci.* 22, 430-440. 10.1016/S1044-7431(03)00029-0 12727441

[DMM052471C27] Mohamad, T., Plante, C. and Brosseau, J.-P. (2021). Toward understanding the mechanisms of malignant peripheral nerve sheath tumor development. *Int. J. Mol. Sci.* 22, 8620. 10.3390/ijms22168620 34445326 PMC8395254

[DMM052471C28] Nishimura, E. K., Suzuki, M., Igras, V., Du, J., Lonning, S., Miyachi, Y., Roes, J., Beermann, F. and Fisher, D. E. (2010). Key roles for transforming growth factor beta in melanocyte stem cell maintenance. *Cell Stem Cell* 6, 130-140. 10.1016/j.stem.2009.12.010 20144786 PMC3437996

[DMM052471C29] Parfejevs, V., Debbache, J., Shakhova, O., Schaefer, S. M., Glausch, M., Wegner, M., Suter, U., Riekstina, U., Werner, S. and Sommer, L. (2018). Injury-activated glial cells promote wound healing of the adult skin in mice. *Nat. Commun.* 9, 236. 10.1038/s41467-017-01488-2 29339718 PMC5770460

[DMM052471C30] Prieto-Granada, C. N., Wiesner, T., Messina, J. L., Jungbluth, A. A., Chi, P. and Antonescu, C. R. (2016). Loss of H3K27me3 expression is a highly sensitive marker for sporadic and radiation-induced MPNST. *Am. J. Surg. Pathol.* 40, 479-489. 10.1097/PAS.0000000000000564 26645727 PMC4882106

[DMM052471C31] Rebecca, V. W., Somasundaram, R. and Herlyn, M. (2020). Pre-clinical modeling of cutaneous melanoma. *Nat. Commun.* 11, 2858. 10.1038/s41467-020-15546-9 32504051 PMC7275051

[DMM052471C32] Schindelin, J., Arganda-Carreras, I., Frise, E., Kaynig, V., Longair, M., Pietzsch, T., Preibisch, S., Rueden, C., Saalfeld, S., Schmid, B. et al. (2012). Fiji: an open-source platform for biological-image analysis. *Nat. Methods* 9, 676-682. 10.1038/nmeth.2019 22743772 PMC3855844

[DMM052471C33] Shakhova, O., Zingg, D., Schaefer, S. M., Hari, L., Civenni, G., Blunschi, J., Claudinot, S., Okoniewski, M., Beermann, F., Mihic-Probst, D. et al. (2012). Sox10 promotes the formation and maintenance of giant congenital Naevi and melanoma. *Nat. Cell Biol.* 14, 882-890. 10.1038/ncb2535 22772081

[DMM052471C34] Wellbrock, C. and Hurlstone, A. (2010). BRAF as therapeutic target in melanoma. *Biochem. Pharmacol.* 80, 561-567. 10.1016/j.bcp.2010.03.019 20350535

[DMM052471C35] Wong, C. E., Paratore, C., Dours-Zimmermann, M. T., Rochat, A., Pietri, T., Suter, U., Zimmermann, D. R., Dufour, S., Thiery, J. P., Meijer, D. et al. (2006). Neural crest-derived cells with stem cell features can be traced back to multiple lineages in the adult skin. *J. Cell Biol.* 175, 1005-1015. 10.1083/jcb.200606062 17158956 PMC2064709

[DMM052471C36] Zhang, X., Lou, H. E., Gopalan, V., Liu, Z., Jafarah, H. M., Lei, H., Jones, P., Sayers, C. M., Yohe, M. E., Chittiboina, P. et al. (2022). Single-cell sequencing reveals activation of core transcription factors in PRC2-deficient malignant peripheral nerve sheath tumor. *Cell Rep.* 40, 111363. 10.1016/j.celrep.2022.111363 36130486 PMC9585487

[DMM052471C37] Zingg, D., Debbache, J., Schaefer, S. M., Tuncer, E., Frommel, S. C., Cheng, P., Arenas-Ramirez, N., Haeusel, J., Zhang, Y., Bonalli, M. et al. (2015). The epigenetic modifier EZH2 controls melanoma growth and metastasis through silencing of distinct tumour suppressors. *Nat. Commun.* 6, 6051. 10.1038/ncomms7051 25609585

[DMM052471C38] Zingg, D., Arenas-Ramirez, N., Sahin, D., Rosalia, R. A., Antunes, A. T., Haeusel, J., Sommer, L. and Boyman, O. (2017). The histone methyltransferase Ezh2 controls mechanisms of adaptive resistance to tumor immunotherapy. *Cell Rep.* 20, 854-867. 10.1016/j.celrep.2017.07.007 28746871

[DMM052471C39] Zingg, D., Debbache, J., Peña-Hernández, R., Antunes, A. T., Schaefer, S. M., Cheng, P. F., Zimmerli, D., Haeusel, J., Calçada, R. R., Tuncer, E. et al. (2018). EZH2-mediated primary cilium deconstruction drives metastatic melanoma formation. *Cancer Cell* 34, 69-84.e14. 10.1016/j.ccell.2018.06.001 30008323

