# Supplementary material for: A novel murine model for sporadic, malignant peripheral nerve sheath tumors, driven by BrafV600E and Pten loss
Source: Dis Model Mech. 2025 Nov 28;18(11):dmm052471. doi: 10.1242/dmm.052471 (PMC12690522; doi:10.1242/dmm.052471)
Supplement: Supplementary information [file dmm-18-052471-s1.pdf]

Figure S1

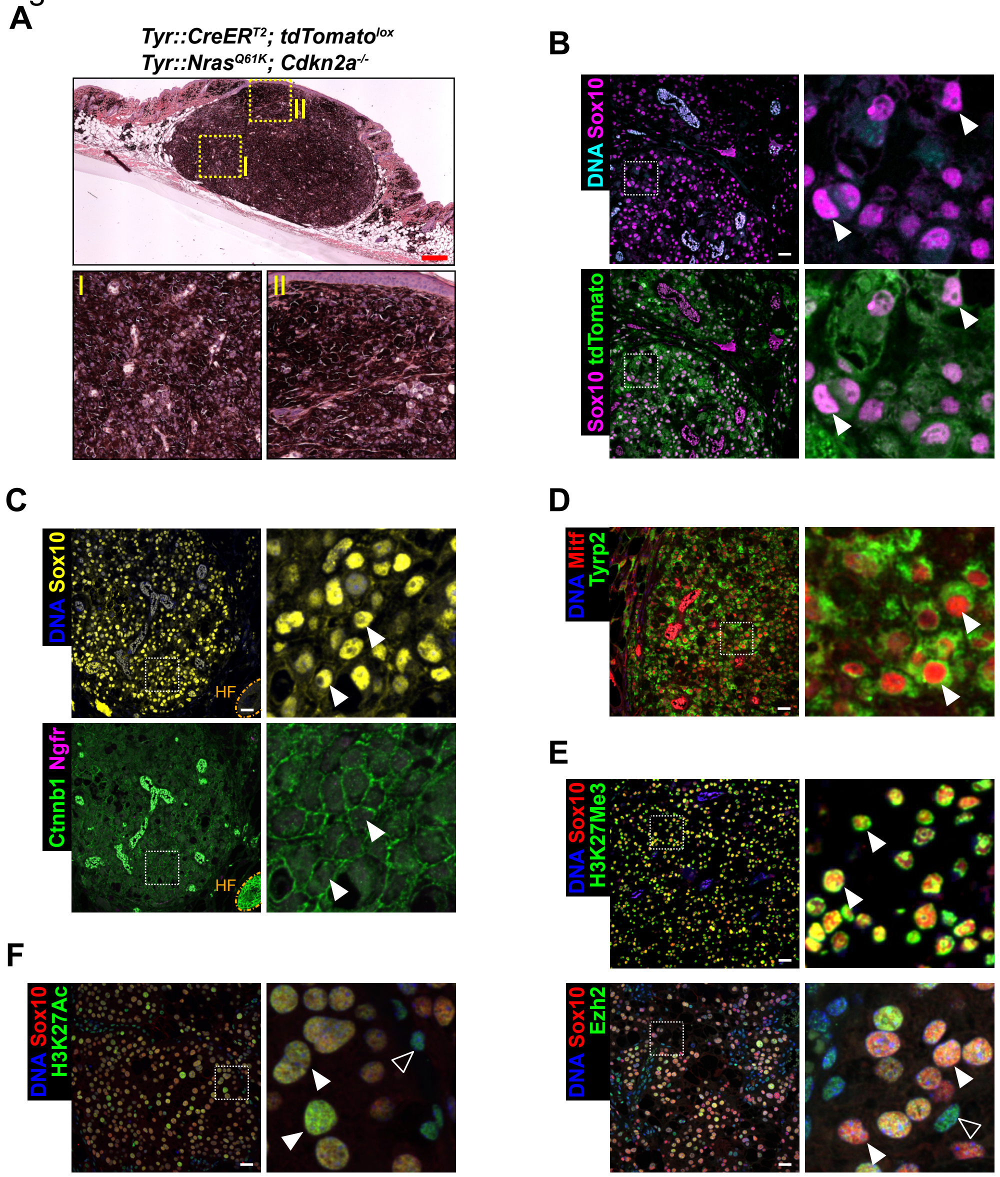

Fig. S1. Established tumors in spontaneous melanoma models show features distinct from skin MPNST.

(A-F) Comparative histology of spontaneous skin tumors from *Tyr::CreER^{T2}; tdTomato^{lox}; Tyr::Nras^{Q61K}; Cdkn2a$^{-/-}$* mice (complementary panel). (A) Hematoxylin-Eosin staining. Scale bars 250 µm. Immunofluorescence staining for (B) Sox10 and tdTomato, (C) Sox10, Ctnnb1 and Ngfr, (D) Mitf and Tyrp2, (E) SOX10, H3K27Me3 and Ezh2, (F) Sox10 and H3K27Ac . (B-F) DNA counterstaining with Hoechst 33342. White arrowheads, melanoma cells, empty arrowheads, stromal cells. Scale bars 25 µm.

Figure S2

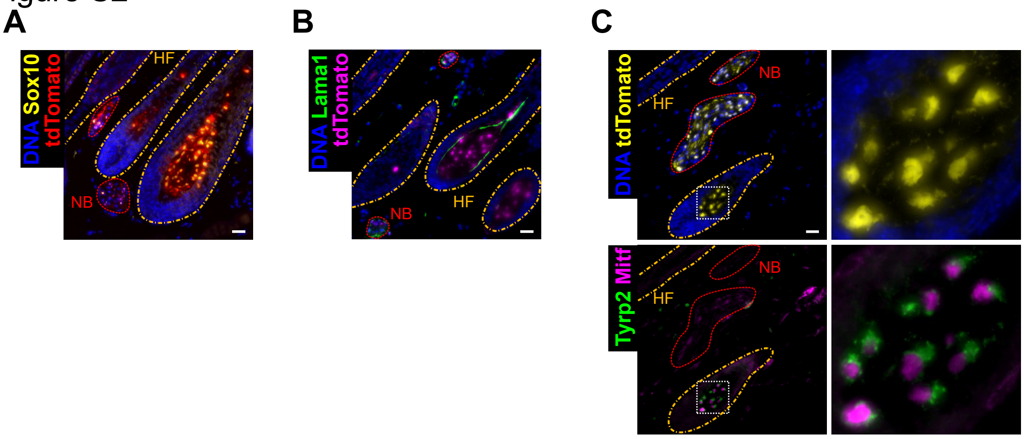

Fig. S2. Complementary histological analysis of murine MPNST

(A-C) HF bulb region including melanocytes and staining for (A) Sox10, tdTomato, (B) Lama1 and tdTomato, (C) tdTomato, and melanocytic markers Mitf and Tyrp2. DNA counterstaining with Hoechst 33342. Orange dashed lines, HFs; red dashed lines, NBs. Scale bars 25 µm.

Figure S3

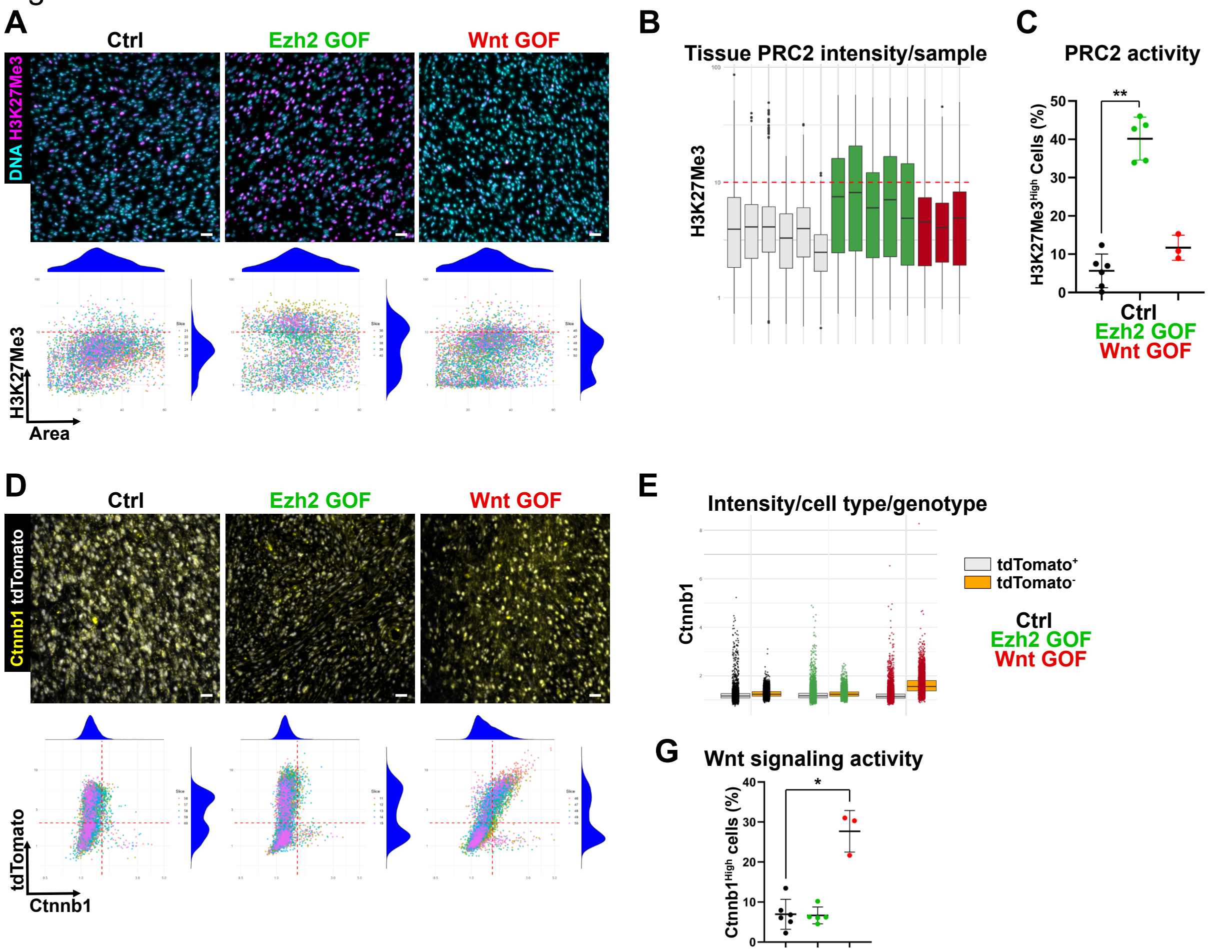

Fig. S3. Validation of Wnt signal activation and PRC2 activity in MPNST.
(A) Representative IF images per genotype depicting PRC2 activity (H3K27Me3) with their respective representation of segmented IF data. (B) Normalized H3K27Me3 intensities per cell aggregated per biological samples (Ctrl n = 6; Ezh2 GOF n = 5; Wnt GOF n = 3). (C) Averaged proportion of H3K27Me3^{High} (Normalized intensity >10) segmented nuclei per image for each biological sample (Ctrl n = 6; Ezh2 GOF n = 5; Wnt GOF n = 3). (D) Representative IF images per genotype depicting Ctnnb1 levels and tumor marker tdTomato with their respective segmented IF data representation. (E) Normalized Ctnnb1 intensities per cell, categorized per tdTomato levels (<2: Stroma or ≥2: Tumor) and genotype (tdTomato Ctrl n = 2; tdTomato Ezh2 GOF n = 2; tdTomato Wnt GOF n = 3). (F) Averaged proportion of Ctnnb1^{High} (Normalized intensity >1.5) segmented nuclei per image for each biological sample (Ctrl n = 6; Ezh2 GOF n = 5; Wnt GOF n = 3). Scale bars 25 μm (A and D). p values were calculated with unpaired nonparametric Mann-Whitney rank test. * $p < 0.05$, ** $p < 0.01$.

Table S1. Genotyping primers

Gene	P1	P2
Braf^{CA}	GCTTGGCTGGACGTAAACTC	-
Braf^{WT}	GCCCAGGCTCTTTATGAGAA	AGTCAATCATCCACAGAGACCT
Cre	CTATCCAGCAACATTTGGGCCAGC	CCAGGTTACGGATATAGTTCATGAC
Ctnnb1$^{\Delta Ex3}$	GACACCGCTGCGTGGACA	GTGGCTGACAGCAGCTTT
Col1A1::LSL-Ezh2^{Y646N}	TTTCTGTGCCATTGCTAGGT	ACTTTCCCTCTTCTGTCAGC
Pten^{lox}	CAAGCACTCTGCGAACTGAG	AAGTTTTTGAAGGCAAGATGC
R26-WT (tdTomato-null)	AAGGGAGCTGCAGTGGAGTA	CCGAAAATCTGTGGGAAGTC
tdTomato^{lox}	CTGTTCCTGTACGGCATGG	GGCATTAAAGCAGCGTATCC
